# Effects of dog-assisted therapy in adults with dementia: a systematic review and meta-analysis

**DOI:** 10.1186/s12888-018-2009-z

**Published:** 2019-01-24

**Authors:** Jessica Hanae Zafra-Tanaka, Kevin Pacheco-Barrios, Walter Andree Tellez, Alvaro Taype-Rondan

**Affiliations:** 1 CRONICAS Center of Excellence for Chronic Diseases, Universidad Peruana Cayetano Heredia, Lima, Peru; 2 Unidad de Investigación para la Generación y Síntesis de Evidencias en Salud, Universidad San Ignacio de Loyola, Lima, Peru; 3 Sociedad Científica de Estudiantes de Medicina Villarrealinos, Universidad Nacional Federico Villarreal, Lima, Peru

**Keywords:** Animal-assisted therapy, Cognitive dysfunction, Dementia, Meta-analysis

## Abstract

**Background:**

Dog-assisted therapy (DAT) is a non-pharmacological intervention based on the interaction between patients and dogs, which has been proposed to help adults with dementia. However, evidence to support it is lacking. Thus, we aim to evaluate the effects of DAT on this population and to assess the certainty of the evidence of the RCTs estimates.

**Methods:**

A systematic search was performed. We included randomized controlled trials (RCTs) and quasi-experimental (QE) controlled studies published up to March 2018, which evaluated the beneficial and deleterious effects of DAT in adults with dementia. Mean differences (MD) or standardized mean differences (SMD) and their 95% confidence intervals (95% CI) were calculated and random effects meta-analyses were performed. Certainty of evidence was assessed for RCTs estimates using the *Grading of Recommendations Assessment, Development, and Evaluation* (GRADE) methodology. The study protocol has been registered in PROSPERO (CRD42018090434).

**Results:**

Ten studies (six RCTs and four QE controlled studies) were eligible for inclusion. Meta-analysis of RCTs showed no effect of DAT in daily life activities (SMD: 0.16; 95% CI: -0.80 to 1.12), depression (SMD: -0.45; 95% CI: -2.81 to 1.91), agitation (SDM: -1.12; 95% CI: -2.67 to 0.43), quality of life (SDM: 0.16; 95% CI: -0.41 to 0.73), and cognitive impairment (SDM: -0.52; 95% CI: -1.33 to 0.30), but it found a beneficial effect in apathy (1 study, *n* = 37, MD: 1.81; 95% CI: 1.26 to 2.36). All outcomes had a very low certainty of evidence according to GRADE methodology.

**Conclusions:**

RCTs evidence of very low certainty suggests that, in adults with dementia, DAT has no effect in daily life activities, depression, agitation, quality of life, and cognitive impairment, although one small study found an apparent beneficial effect in apathy. More well-designed and correctly reported studies are needed in order to provide a conclusion.

**Trial registration:**

CRD42018090434 (PROSPERO).

**Electronic supplementary material:**

The online version of this article (10.1186/s12888-018-2009-z) contains supplementary material, which is available to authorized users.

## Introduction

Animal-assisted therapies (AAT) are interventions in which animals participate as an integral part to improve specific outcomes in the patient [1]. Dog-assisted therapies (DAT) is a subtype of AAT in which patients interact with dogs [1]. This interaction can include diverse activities such as petting, brushing, feeding, playing with, strolling with, or talking to the dog. DAT has been described as promising in helping people with diverse conditions, especially psychiatric conditions and cognitive disorders such as dementia [2].

Regarding AAT and its effects on dementia, two systematic reviews have been recently published: one systematic review found 32 studies that addressed AAT, from which 27 used dogs and 8 of these were randomized controlled trials (RCTs); and concluded that AAT is effective in reducing the behavioral and psychological symptoms of dementia [3]. Another systematic review that evaluated the benefits of AAT for cognitive impairment found ten studies (five RCTs and five quasi-experimental [QE] studies) up to June 2017, all of which included dogs, either alone or accompanied by other animals. This systematic review found statistically significant effects of DAT in depression and agitation. However, this is based on a meta-analyses that combined RCTs and non-RCTs, which is not currently suggested [4].

Although these systematic reviews suggest that DAT has some benefits in persons with dementia, they tend to mix results from RCTs and other study designs, which precludes an adequate evaluation of the role of confounding variables. Moreover, these reviews do not assess the certainty of evidence which is necessary when making health care decisions. To evaluate the certainty, we will use a systematic framework called *Grading of Recommendations Assessment, Development, and Evaluation* (GRADE) methodology [5]. GRADE has been increasingly used by different institutions devoted to clinical practice guidelines development and decision making given that it allows reproducibility in the assessment of certainty of evidence [6]. Thus, using GRADE will help us to correctly assess the current confidence in the final estimates and therefore, it will help us formulate recommendations and future studies in the topic. Thus, the aim of this systematic review was to search for RCTs and QE controlled studies in order to evaluate the effects of DAT in adults with dementia and to assess the certainty of the evidence for RCTs estimates using the GRADE methodology.

## Methods

We performed a systematic review. The study protocol has been registered at PROSPERO, number CRD42018090434.

### Literature search and study selection

For this systematic review, we included all RCTs and QE controlled studies that directly evaluated any beneficial or adverse effect of DAT in adults with dementia. We excluded those studies for which full-text could not be accessed.

A literature search was performed in two steps: 1) a systematic review of three databases, and 2) a review of all documents that have cited any of the studies included in step 1. The complete search strategy is available in Additional file 1.

For the first step, we performed a literature search in three databases: Medline, Central Cochrane Library, and Scopus. No restrictions in language or publication date were employed. The last updating search was run on March 2018. Duplicated records were removed using Endnote software. After that, titles and abstracts were screened in order to identify potentially relevant articles for inclusion. Lastly, these potential relevant articles were full-text assessed in order to evaluate their eligibility. The complete list of excluded articles at this full-text stage is available in Additional file 2.

For the second step, we reviewed all documents that have cited any of the studies included in step 1 using Google Scholar (http://scholar.google.com) and collected all articles that fulfilled inclusion and exclusion criteria. The selection process, in step 1 and step 2, was performed independently by two reviewers, and disagreements were resolved through a discussion between all authors.

### Data extraction

Two independent researchers extracted the following information from each of the included studies into a Microsoft Excel sheet: author, year of publication, title, population (inclusion and exclusion criteria), setting, intervention (length, frequency, and activities), comparator (length, frequency, and activities), time of follow-up, and effects of DAT in all included outcomes. In the cases of disagreements, the full-text articles were reviewed again by the researchers in order to correct the mistakes.

### Study quality and certainty of evidence

To assess the risk of bias of RCTs, we used the Cochrane Risk of Bias Tool [7], with the following exception: given that it was not possible to blind the participants for this intervention, this item was not considered for the risk of bias evaluation. To assess the risk of bias of QE controlled studies, we used the Methodological Index for Non-randomized Studies (MINORS) tool [8]. In order to classify in low, high, and unclear risk of bias we followed the instructions stated in the Cochrane handbook for systematic reviews of interventions for RCTs [9]. For QE controlled studies, the MINORS tool considered three possible scores for each item from 0 to 2: 0 for not reported information, 1 for information reported inadequately, and 2 for well-reported information. We considered a low risk of bias when the information was well reported, high risk of bias when it was not reported, and unclear risk of bias when it was reported inadequately [8]. Moreover, for the overall risk of bias assessment using the MINORS tool, we considered that scores less than 16 points indicated a high risk of bias and from 16 to 24 points indicated low risk of bias [10].

To assess the certainty of the evidence, understood as the certainty of the evidence regarding the intervention effects, we used the GRADE methodology [11], which is based in the following criteria: risk of bias, inconsistency, indirectness, and imprecision. Given that this methodology is currently focused on RCTs and that the GRADE working group has not reached a consensus of whether to combine results from randomized and non-randomized trials, we only present this evaluation for RCTs [11].

### Statistical analyses

We performed meta-analyses in order to summarize studies that have evaluated similar outcomes. When outcomes were measured using different scales across studies, we calculated standardized mean differences (SMD) to compare and meta-analyze these studies. For outcomes that were evaluated in only one study, we present the mean difference (MD) as no meta-analysis was performed.

For studies that had been measured more than once (before and after the intervention or repeated measures), as for those that were only measured at the end of the intervention, we only presented the final measurement of each outcome, and we only considered this measurement to perform the meta-analyses, as suggested in the Cochrane Handbook [12] given that there were participants who were lost to follow-up.

We decided to present results separately according to study design (RCTs vs QE controlled studies), given the differences in certainty of evidence between these two types of studies, and that the evaluation of the certainty of evidence using the GRADE methodology was performed only on RCTs.

We assessed heterogeneity using an I^2^ statistical and we considered that heterogeneity might not be important when I^2^ < 40% [12]. We consider appropriate to use random-effects models due to the overall heterogeneity evaluation (in population, intervention, and comparators [13]. Publication bias was not statistically assessed since the number of studies pooled for each meta-analysis was less than ten [14]. The data was processed using Stata v14.0 software.

## Results

### Studies characteristics

**Fig. 1:**
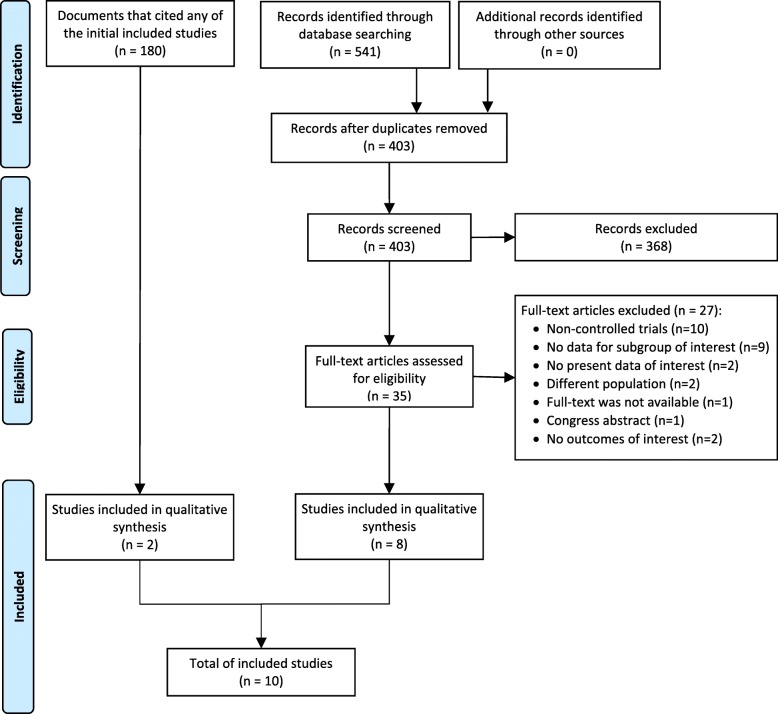
Flow diagram (study selection)

In the systematic review of three databases, we found a total of 541 titles. We removed 138 duplicates and screened a total of 403 titles, 35 studies were evaluated in full-text and eight were included [15–21]. Additionally, we evaluated 180 documents that have cited any of the eight studies included, from which two new studies were included [22, 23] (Fig. 1).

Regarding the included studies, six were RCTs [15, 16, 20, 22, 23] –including one cluster randomized trial [20] and one cross-over RCT [22]–, while four were QE controlled studies [17–19, 21]. The number of participants ranged from 32 to 79 in RCTs, and from 10 to 54 in QE controlled studies.

Regarding the study setting, four studies were performed in daycare centers, five in nursing homes and one in an assisted living facility; however, intervention setting was heterogeneous (spaces specially designed for the study, nursing homes, or special assisted facilities). Regarding dementia diagnosis for inclusion criteria, five studies used the Mini-Mental State Examination (MMSE) with different cut-off points, two studies included patients that had the diagnosis of dementia in the clinical history, and three used other criteria; in addition, studies included diverse severity degree of dementia (i.e. very mild to severe dementia). Regarding the intervention, it consisted on DAT sessions that involved interaction with dogs in an individual or group session and lasted from 10 to 90 min, during a time lapse of 2 weeks to 8 months, and a frequency from one to three times per week. Interventions were heterogeneous and could include several activities such as greeting the dog, playing with the dog, talking to the dog, recalling personal events through the dog, among others. Regarding the control group, it received either usual care, human-visits, reminiscence therapy, or active comparator using plush-dogs (Additional file 3).

#### Regarding outcome measurements

Four studies assessed daily life activities: three studies [15, 16, 21] used the Barthel index and one [17] used the activities of daily living (N-ADL) score; for all scales, a higher score means more independence.

Seven studies assessed depression: five studies [15, 16, 19–21] used the Cornell Scale for Depression in Dementia (CSDD), one [18] used the Dementia Mood Assessment Scale (DMAS) and one [23] used the Multidimensional Observation Scale for Elderly Subjects (MOSES) [24]; for all scales, a higher score means more chance of depression.

Five studies assessed agitation: two studies used the complete Cohen-Mansfield Agitation Inventory (CMAI) [18, 19], two studies used the short CMAI [16, 22], and one study [20] used the Brief Agitation Rating Scale (BARS); for all scales, a higher number of points means more agitation.

Four studies assessed QoL: three studies used the Quality of Life in Late-Stage Dementia (QUALID) [20, 21] and one study used the Quality of Life in Alzheimer Disease (QOL-AD) [23]; for the QUALID scale less points mean better quality of life while for the QOL-AD scale more points mean better quality of life, for this reason we inverted the results from the study that used the QOL-AD.

Three studies assessed cognitive function: two [17, 21] used the (MMSE) for which a higher number of points mean better cognitive function and one of them [15] used the Alzheimer disease assessment scale (ADAS), for which a higher number of points means less cognitive function; for this reason, we inverted that scale so that a higher number of points for cognitive function would reflect better function.

One RCT evaluated Apathy using the Zimmerman’s short version of the Apathy Evaluation Scale (AES), for which a lower score reflects more apathy [25, 26].

One study evaluated different dimensions of the MOSES [24]: self-care, disorientation, irritability, and withdrawal, for which a higher score reflects poorer outcomes.

### Risk of bias

**Fig. 2:**
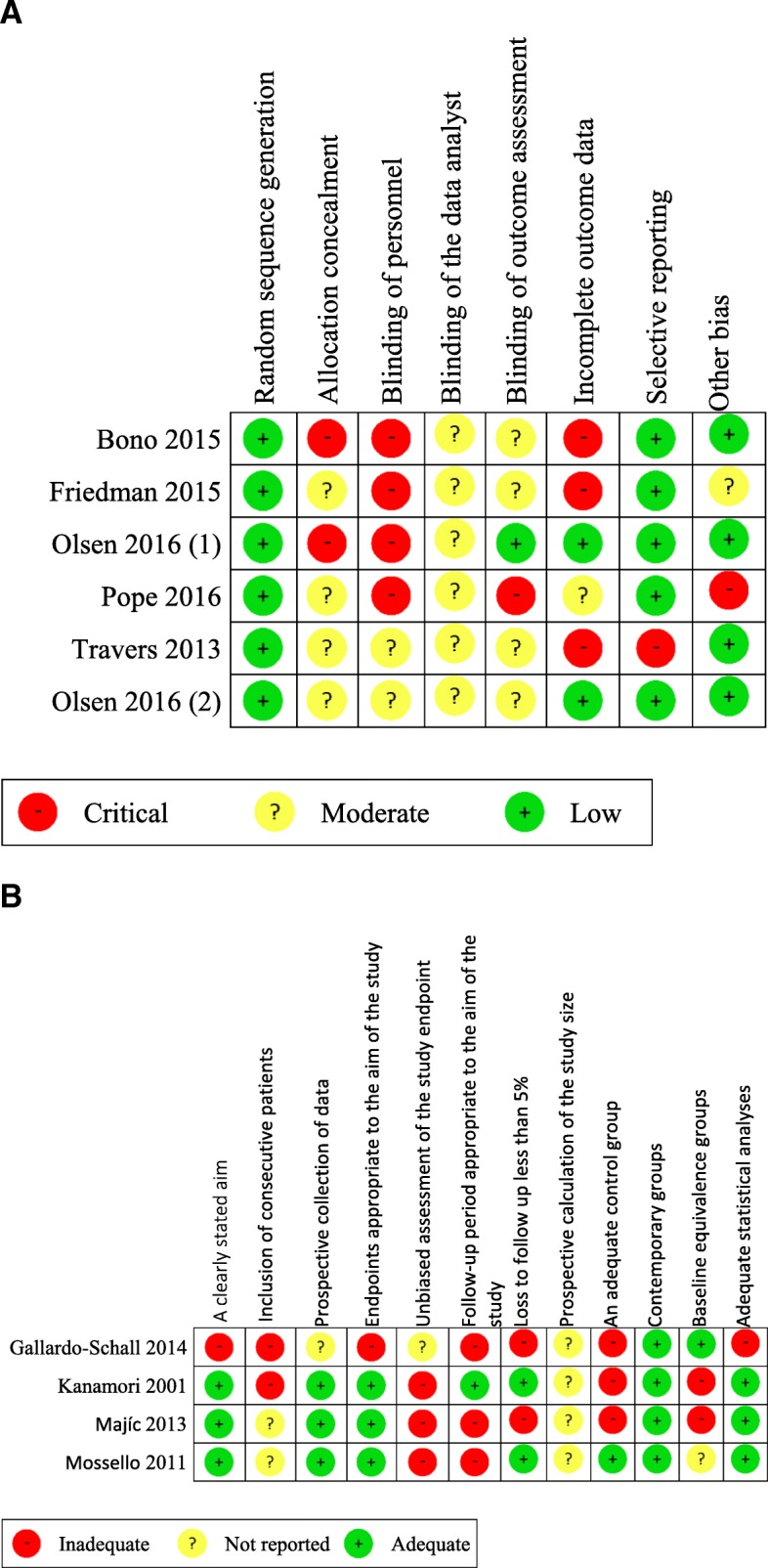
Risk of bias. **a**. Risk of bias of RCTs (Olsen (1): Olsen C, et al. 2016 [32]. Olsen (2): Olsen C, et al [20]). **b**. Risk of bias of QE studies

The risk of bias for six RCTs was assessed using the Cochrane tool. All RCTs had problems when reporting allocation concealment, blinding of personnel and blinding of the data analyst; and five had problems with blinding of outcome assessment (Fig. 2a).

The risk of bias for four QE controlled studies was assessed using the MINORS tool. All studies had problems when reporting the calculation of sample size, with the unbiased assessment of the study endpoint, and the inclusion of consecutive patients (Fig. 2b).

### Effects on outcomes

**Fig. 3:**
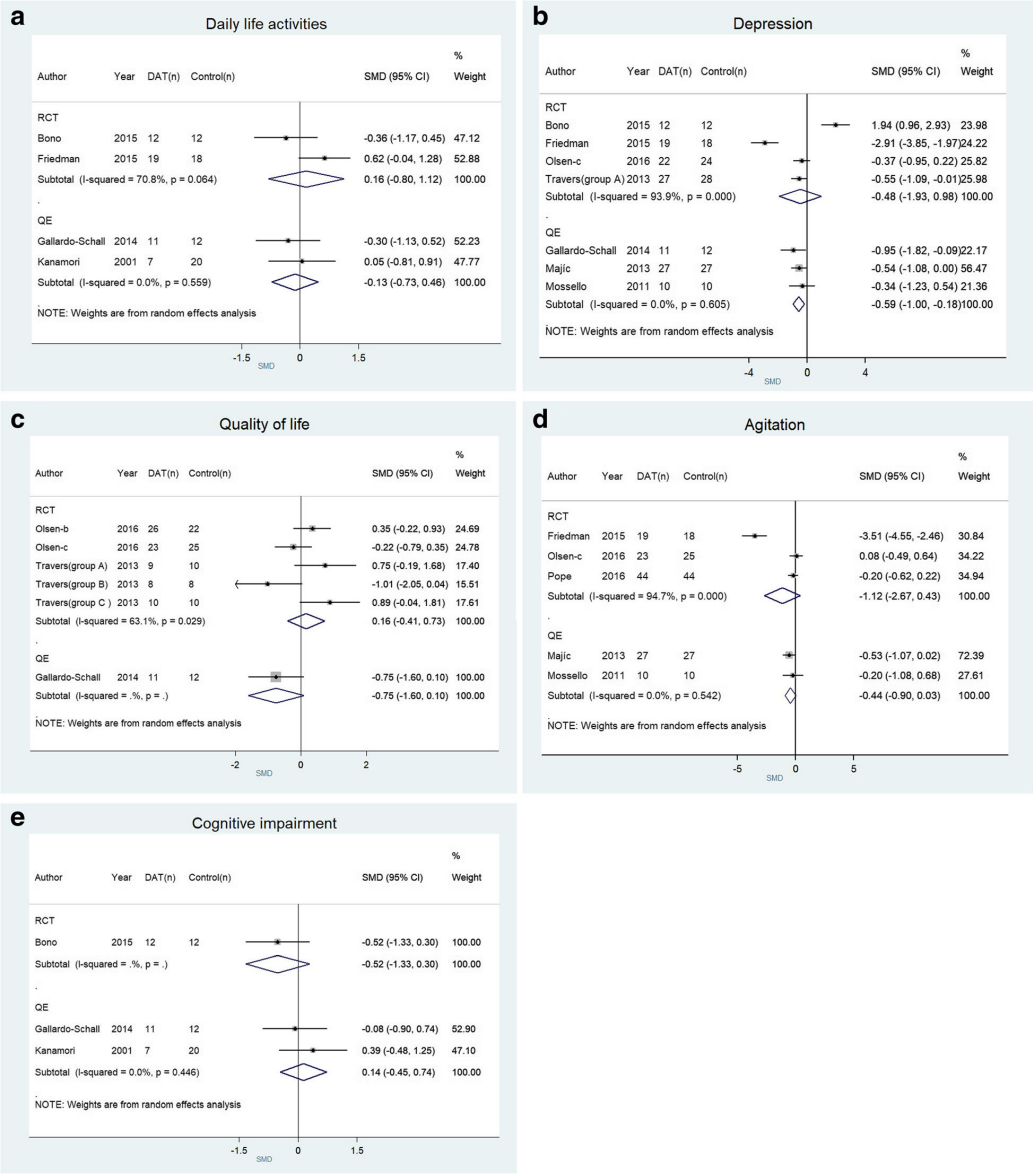
Forest plot on dog-assisted therapies for assessed outcomes. **a** Forest plot on dog-assisted therapies for the improvement in daily life activities. Right favors DAT, left favors control. **b** Forest plot on dog-assisted therapies for the improvement in depression. Right favors control, left favors DAT. **c** Forest plot on dog-assisted therapies for the improvement in quality of life. Right favors DAT, left favors control. **d** Forest plot on dog-assisted therapies for the improvement in agitation. Right favors control, left favors DAT. **e** Forest plot on dog-assisted therapies for the improvement in cognitive impairment. Right favors DAT, left favors control

When pooling RCTs, we found no effect of DAT in daily life activities (2 RCTs, SMD: 0.16; 95% CI: -0.80 to 1.12) (Fig. 3a), depression (4 RCTs, SMD: -0.48; 95% CI: -1.93 to 0.98) (Fig. 3b), QoL (5 RCTs, SDM: 0.16; 95% CI: -0.41 to 0.73) (Fig. 3c), agitation (3 RCTs, SDM: -1.12; 95% CI: -2.67 to 0.43) (Fig. 3d), and cognitive impairment (1 RCT, SDM: -0.52; 95% CI: -1.33 to 0.30) (Fig. 3e). However, we found an effect in apathy (1 RCT, SMD: 2.10; 95% CI: 1.29 to 2.91). No RCT evaluated adverse effects of DAT.

When pooling QE controlled studies, we found no effect of DAT in daily life activities (2 QE studies, SMD: -0.13; 95% CI: -0.73 to 0.46) (Fig. 3a), QoL (1 QE study, SDM: -0.75; 95% CI: -1.60 to 0.10) (Fig. 3c), agitation (2 QE studies, SDM: -0.44; 95% CI: -0.90 to 0.03) (Fig. 3d), cognitive impairment (2 QE studies, SDM: 0.14; 95% CI: -0.45 to 0.74) (Fig. 3e), self-care (1 QE study, MD: 0.01; 95% CI: -3.23 to 3.43), disorientation (1 QE study, MD: -0.9; 95% CI: -4.34 to 2.54), irritability (1 QE study, MD: -1.1; 95% CI: -3.08 to 0.88), and withdrawal (1 QE study, MD: -0.6; 95% CI: -3.17 to 1.97). However, when pooling QE controlled studies, DAT showed an effect in depression (3 QE studies, SMD: -0.59; 95% CI: -1.00 to − 0.18) (Fig. 3b). No QE controlled study evaluated adverse effects of DAT.

### Certainty of evidence

We used the GRADE methodology to assess the certainty of evidence from RCTs. We found that, for the outcomes studied (daily life activities, depression, QoL, agitation, cognitive impairment, and apathy), the evidence was of very low certainty. This was mainly due to the risk of bias, inconsistency (I^2^ was higher than 40% and the 95% CI of the studies estimates did not overlap), and imprecision (due to small sample sizes) (Table 1).

**Table 1:** Summary of findings to evaluate the certainty of the evidence, using the GRADE methodology

Outcomes	Anticipated absolute effects^*^ (95% CI)	Relative effect (95% CI)	№ of participants and studies (I: intervention, C: control)	Certainty of the evidence (GRADE)	Comments
	Risk with Animal-assisted therapy				
Activities of daily living	SMD 0.16 SD more (0.80 lower to 1.12 more)	–	I 31 C 30 (2 RCTs)	⨁◯◯◯ VERY LOW^a,b,c^	We are uncertain about the effect of DAT on activities of daily living.
Depression	SMD 0.48 SD lower (1.93 lower to 0.98 higher)	–	I 80 C 82 (4 RCTs)	⨁◯◯◯ VERY LOW^a,b,c,d^	We are uncertain about the effect of DAT on depression.
Agitation	SMD 1.12 SD lower (2.67 lower to 0.43 higher)	–	I 86 C 87 (3 RCTs)	⨁◯◯◯ VERY LOW^a,b,c^	We are uncertain about the effect of DAT on agitation.
Quality of life	SMD 0.16 SD higher (0.41 lower to 0.73 higher)	–	I 76 C 75 (3 RCTs)	⨁◯◯◯ VERY LOW^b,c,e^	We are uncertain about the effect of DAT on quality of life.
Cognitive impairment	SMD 0.52 SD lower (1.33 lower to 0.30 higher)		I 12 C 12 (1 RCT)	⨁◯◯◯ VERY LOW^a,c^	We are uncertain about the effect of DAT on cognitive impairment.
Apathy	SMD 2.10 SD higher (1.29 higher to 2.91 higher)		I 19 C 18 (1 RCT)	⨁◯◯◯ VERY LOW^a,c^	We are uncertain about the effect of DAT on apathy.

Explanations

a. Blinding of outcome assessment was not detailed in the publication.

b. Point estimates vary widely, and confidence intervals do not overlap.

c. Sample sizes were small (< 400).

d. Risk of bias due to inadequate measurement of outcomes.

e. RCTs were not blinded. Selective reporting possibly occurred in one of the included RCT

## Discussion

### Summary of the results

We included ten studies (six RCTs and four QE controlled studies) that have evaluated the effects of DAT in people with dementia. These studies were heterogeneous, had a low sample size, and presented a high risk of bias. Pooled RCTs did not find benefits in daily life activities, depression, agitation, QoL, or cognitive impairment; however, the only RCT that evaluated apathy found a potentially beneficial effect. Certainty of evidence for RCTs was very low. Pooled QE controlled studies did not find benefits in daily life activities, agitation, QoL, cognitive impairment, self-care, disorientation, irritability, or withdrawal. On the other hand, a potential beneficial effect in depression was found.

### Comparison with other studies

The four studies that evaluated the effect of DAT in daily life activities, as well as the meta-analyses of two RCTs and of two QE controlled studies, found no benefit of DAT in daily life activities. Similarly, a systematic review published in 2018 that evaluate the effect of AAT on cognitive impairment [4] meta-analyzed three studies (two RCTs and one QE study), all of which were included in our review [15, 17, 21], and found no effect of AAT on daily life activities.

Regarding the effect of DAT on depression, our meta-analysis of four RCTs found no benefit, while our meta-analysis of three QE controlled studies showed a slight effect. However, given the methodological limitations of QE studies in controlling important confounding variables [27], results of these studies must be taken with extreme caution when RCTs do not show such effect. In addition, RCTs were affected by imprecision and the methodological limitations of these RCTs (small sample size, heterogeneity of the control group intervention and outcome measure), and the QE design. Thus, high-quality RCTs are needed to stablish the true beneficial effects of DAT in this outcome. Conversely, a meta-analysis published in 2018 [4] pooled four studies (two RCTs and two non-controlled QE studies, from which the two RCTs [16, 20] were included in our analysis) and observed a beneficial effect of AAT in depression in patients with dementia when pooling QE studies and RCTs.

Our meta-analyses on three RCTs and on two QE controlled studies found no benefit of DAT in agitation. A previous meta-analysis [4] found lower agitation in the DAT group. The previous systematic review [4] published in 2018 that evaluated the effect of AAT on cognitive impairment, meta-analyzed four studies (two RCTs and two QE studies), from which three studies [16, 18, 20] were included in our analysis (the other were a non-controlled QE study). However, this result was obtained from a pooled effect from RCTs and QE studies, for which one QE study without a control group was included [28]. All these facts confer low certainty on the results previously reported.

In all the studies that evaluated QoL [20, 21, 23], no improvement was found in the DAT group; however, it was a very broad construct with many variables that could affect it, and the studies were heterogeneous in settings and interventions. Nevertheless, considering that these patients have no therapeutic options to modify the disease, the improvement in the QoL becomes a critical outcome, making this an important outcome that should be evaluated in future studies.

We found an RCT [16] that showed an effect on apathy in favor of DAT group, measured with the short version of the AES (range from 7 to 28 points, lower scores indicate higher apathy). The study showed that final apathy score was higher in the DAT group than in the control group. However, the results remain uncertain regarding the small sample size of the study (19 DAT and 18 control participants).

### Certainty of evidence and implications for clinical practice

In order to delineate the rationale for going from evidence to recommendation, we will explore the determinants raised by GRADE: balance of desirable and undesirable outcomes, certainty of evidence, preferences of patients and relatives, and resource implications [29].

Regarding the balance of desirable and undesirable outcomes, our pooled meta-analysis on RCTs showed evidence that DAT has no important beneficial effects except for a potential benefit on apathy found in a small RCT. On the other hand, harms of DAT (including fear, anxiety, allergies, bites, falls, infections, and musculoskeletal illness) were not clearly reported in the included studies.

Regarding the certainty of evidence, included studies have a high risk of bias. It is worrying that all the RCTs have a moderate or critical risk of bias for blinding of personnel and blinding of the outcome assessment, which is feasible to perform. We found that all the included studies have a small sample size (< 100 patients) generating imprecision in the effect estimate; moreover, the studies did not calculate the adequate sample size to detect differences on the main outcomes. For example, for the case of depression, in order to find an MD of three points in the CSDD, which has been found to be clinically relevant in previous studies [16, 30], with a power of 80% and an alfa of 5%, a minimum of 204 patients should have been included.

Regarding patient and relatives’ preferences, some of them could be afraid or would not like to work with dogs. On the other hand, the acceptability of health center staff could be adequate because the DAT intervention is usually outsourced to external institutions. Regarding infrastructure, DAT should take place in adequate settings; wide, open spaces. With respect to human and non-human resources; personnel who knows how to handle dogs and an adequately trained dog are needed. Even though most of the studies did not report information on costs of DAT implementation, a previous study reported that around 8000USD are needed to take care of a dog and provide it with adequate housing, food, and veterinary care during its entire life [31]. This can represent a huge amount of money for some health systems.

To sum up, we found no clear evidence of any benefit, and null evidence of possible harms. Given that (irrespectively from resource use, patient preference, feasibility, and acceptability), DAT should not be used routinely as a therapy for patients with dementia.

### Limitations and strengths

Due to the small number of heterogeneous studies that were analyzed, it can be argued that meta-analyses are not comparing similar studies. However, since summarized effect estimates are needed for decision-making, we found meta-analyses useful to give a better overwatch of the results. Besides, the present meta-analysis has the recognized limitations in the primary studies, as insufficient detail about the outcome evaluation, inclusion criteria, intervention, and what did the control group receive.

However, this systematic review has important strengths: it followed the PRISMA statement and was inscribed in the PROSPERO database. In addition, we performed a comprehensive search strategy across multiple databases, without language restriction, and across articles that cited each of the found studies; which allowed us to find all articles found in previous systematic reviews [3, 4], and others that were not found in these reviews. Lastly, we performed an evaluation of the certainty of evidence using the GRADE methodology. These strengths allow us to report the state of the art on the RCTs and QE controlled research in DAT; along with the certainty of evidence and implications for implementation.

## Conclusion and research recommendations

We found 6 RCTs and 4 QE controlled studies that have evaluated the effects of DAT in persons with dementia. We found very low certainty of evidence suggesting that DAT has no effect on daily life activities, depression, QoL, agitation, and cognitive impairment; although the only RCT that evaluated apathy found an apparent benefic effect. No RCT assessed the harms of this intervention. Given that included studies had a small sample size and important risk of bias, and that the certainty in evidence is very low, more RCTs are needed to evaluate the benefits and risks of DAT in patients with dementia. These studies need to be adequately reported, minimize the risk of bias, describe adverse effects, and be more detailed in the description of interventions in DAT and control groups.

## Additional files

Additional file 1: Search strategy. (DOCX 14 kb)

Additional file 2: Studies that were evaluated in full-text, and were excluded. (DOCX 16 kb)

Additional file 3: Study characteristics of individual studies. (DOCX 19 kb)
