# Supplementary material for: Effects of dog-assisted therapy in adults with dementia: a systematic review and meta-analysis
Source: BMC Psychiatry. 2019 Jan 24;19:41. doi: 10.1186/s12888-018-2009-z (PMC6345014; doi:10.1186/s12888-018-2009-z)
Supplement: Supplementary file 1 — Search strategy. (DOCX 14 kb) [file 12888_2018_2009_MOESM1_ESM.docx]

## Supplementary material 1: search strategy

|  |  | Date | Results |
| --- | --- | --- | --- |
| Pubmed | (dementia[Mesh] OR dementia[tiab] OR “Alzheimer Disease”[Mesh] OR (Alzheimer*[tiab] AND disease[tiab]) OR “Cognitive Dysfunction”[Mesh] OR “cognitive impairment”[tiab] OR "Neurocognitive Disorders"[Mesh] OR "Neurocognitive Disorders"[tiab] OR "cognitively impaired"[tiab] OR “cognitive decline”[tiab]) AND ("Pets"[Mesh] OR “pet therapy”[tiab] OR “Animal Assisted Therapy"[Mesh] OR "Animal Assisted"[TIAB] OR animal-assisted[tiab] OR "Bonding, Human-Pet"[Mesh] OR “Animal Human Bonding”[TIAB] OR “canine-assisted”[TIAB] OR “dog-assisted”[TIAB] OR companion animal*[tiab] OR “pets as therapy”[tiab] OR “pet as therapy”[tiab] OR ((dog[tiab] OR animal[tiab] OR pet[tiab]) AND visitation[tiab])) | March 2018 | 88 |
| Scopus | TITLE-ABS-KEY ((dementia OR (Alzheimer* W/1 disease) OR “Cognitive Dysfunction” OR “cognitive impairment” OR "Neurocognitive Disorders" OR “cognitive decline”) AND (“pet therapy” OR "Animal Assisted" OR "Bonding, Human-Pet" OR “Animal Human Bonding” OR “canine-assisted” OR “dog-assisted” OR “companion animal*” OR pets as therapy OR pet as therapy OR ((dog OR animal OR pet) AND visitation))) | March 2018 | 160 |
| CENTRAL | 1. MeSH descriptor dementia, this term only 2. MeSH descriptor Alzheimer Disease, this term only 3. MeSH descriptor Cognitive Dysfunction, this term only 4. MeSH descriptor Neurocognitive Disorders, this term only 5. Dementia:ti,ab or (Alzheimer*:ti,ab and disease:ti,ab) or cognitive impairment:ti,ab or Neurocognitive Disorders:ti,ab or cognitively impaired:ti,ab or cognitive decline:ti,ab 6. MeSH descriptor Pets, this term only 7. MeSH descriptor Animal Assisted Therapy, this term only 8. MeSH descriptor Bonding, Human-Pet, this term only 9. pet therapy:ti,ab or animal assisted:ti,ab or animal-assisted:ti,ab or animal human bonding:ti,ab or canine-assisted:ti,ab or dog-assisted:ti,ab or companion animal*:ti,ab or pet as therapy:ti,ab or pets as therapy:ti,ab 10. dog:ti,ab or animal:ti,ab or pet:ti,ab and visitation:ti,ab 11. #1 or #2 or #3 or #4 or #5 12. #6 or #7 or #8 or #9 or #10 13. 13 (final search term):  **#11 and #12** | March 2018 | 293 |
