# Supplementary material for: Effects of dog-assisted therapy in adults with dementia: a systematic review and meta-analysis
Source: BMC Psychiatry. 2019 Jan 24;19:41. doi: 10.1186/s12888-018-2009-z (PMC6345014; doi:10.1186/s12888-018-2009-z)
Supplement: Supplementary file 2 — Studies that were evaluated in full-text, and were excluded. (DOCX 16 kb) [file 12888_2018_2009_MOESM2_ESM.docx]

## Supplementary material 2: Studies that were evaluated in full-text, and were excluded

| **N** | **Author** | **Year** | **Title** | **Reason for exclusion** |
| --- | --- | --- | --- | --- |
| 1 | Antonelli | 2012 | Animal-assisted activities: Effects on the subjective well-being of elderly women attending a day care service | No data for the subgroup of patients with dementia |
| 2 | Churchill | 1999 | Using a therapy dog to alleviate the agitation and desocialization of people with Alzheimer's disease | No control group |
| 3 | Folch | 2016 | Effectiveness of dog-assisted therapy in the elderly. A preliminary study | Different population: elderly patients without dementia |
| 4 | Haughie | 1992 | An Evaluation of Companion Pets with Elderly Psychiatric Patients | No data for the subgroup of patients with dementia |
| 5 | Holthoff | 2013 | Dog-assisted therapy for people with dementia: a randomized, controlled trial | It´s an abstract from a congress |
| 6 | Kawamura | 2007 | Long-term evaluation of animal-assisted therapy for institutionalized elderly people: A preliminary result | No control group |
| 7 | Kongable | 2013 | The effects of pet therapy on the social behavior of institutionalized Alzheimer's clients | Full-text was not found |
| 8 | Kramer | 2009 | Comparison of the effect of human interaction, animal-assisted therapy, and AIBO-assisted therapy on long-term care residents with dementia | Does not assess outcomes of interest |
| 9 | Lutwack-Bloom | 2005 | Effects of Pets versus People Visits with Nursing Home  Residents | Different population: elderly patients |
| 10 | Marx | 2010 | The impact of different dog-related stimuli on engagement of persons with dementia | No control group |
| 11 | McCabe | 2002 | Resident dog in the Alzheimer's special care unit | No data for the subgroup of patients with dementia |
| 12 | Menna | 2012 | Evaluation of social relationships in elderly by animal-assisted activity | No data for the subgroup of patients with dementia |
| 13 | Menna | 2016 | Evaluation of the efficacy of animal-assisted therapy based on the reality orientation therapy protocol in Alzheimer's disease patients: a pilot study | No control group |
| 14 | Moretti | 2011 | Pet therapy in elderly patients with mental illness | No data for the subgroup of patients with dementia |
| 15 | Nordgren | 2014 | Animal-Assisted Intervention in Dementia: Effects on Quality of Life | No control group |
| 16 | Nordgren | 2014 | Effects of dog-assisted intervention on behavioural and psychological symptoms of dementia | No data of interest (no SD) |
| 17 | Olsen | 2016 | Engagement in elderly persons with dementia attending animal-assisted group activity | Does not assess outcomes of interest |
| 18 | Püllen | 2013 | Animal-assisted therapy for demented patients in acute care hospitals | No control group |
| 19 | Quibel | 2017 | Evaluation of the animal-assisted therapy in Alzheimer's disease | It´s a narrative review |
| 20 | Richeson | 2003 | Effects of animal-assisted therapy on agitated behaviors and social interactions of older adults with dementia | No control group |
| 21 | Sellers | 2006 | The evaluation of an animal assisted therapy intervention for elders with dementia in long-term care | No control group |
| 22 | Sollami | 2017 | Pet therapy: an effective strategy to care for the elderly?  An experimental study in a nursing home | No data for the subgroup of patients with dementia |
| 23 | Thodberg | 2016 | Behavioral responses of nursing home residents to visits from a person with a dog, a robot seal or a toy cat | No data for the subgroup of patients with dementia |
| 24 | Thodberg | 2016 | Therapeutic effects of dog visits in nursing homes for the elderly | No data for the subgroup of patients with dementia |
| 25 | Tournier | 2017 | Animal-Assisted intervention in dementia: Effects on neuropsychiatric symptoms and on caregivers' distress perceptions | No control group |
| 26 | Walsh | 1995 | The effects of a ‘pets as therapy’ dog on persons with dementia in a psychiatric ward | No data of interest (no mean and SD per group) |
| 27 | Zisselman | 1996 | A pet therapy intervention with geriatric psychiatry inpatients | No data for the subgroup of patients with dementia |
