# Supplementary material for: Effects of dog-assisted therapy in adults with dementia: a systematic review and meta-analysis
Source: BMC Psychiatry. 2019 Jan 24;19:41. doi: 10.1186/s12888-018-2009-z (PMC6345014; doi:10.1186/s12888-018-2009-z)
Supplement: Supplementary file 3 — Study characteristics of individual studies. (DOCX 19 kb) [file 12888_2018_2009_MOESM3_ESM.docx]

## Supplementary material 3: study characteristics of individual studies

| Author, year (country) | Design | Population, age, dementia definition, and other inclusion/exclusion criteria | Number of patients* | Intervention | Control | Follow-up |
| --- | --- | --- | --- | --- | --- | --- |
| Olsen (1) 2016 (Norway) | Cluster RCT | - Outpatients - Age: ≥65 years old, I 81.7; C 84.0 - Dementia: MMSE score<25, CDRS 1 a 2 (I 87.8% C 94.0%) - Inclusion: - - Exclusion: fear of dogs or with a dog allergy | I: 41, C: 38 | - 30-min sessions twice per week for 12 weeks. - Setting: adapted day-care centers. - Activities: Petting the dog, brushing the dog, feeding the dog a treat, or throwing a toy for the dog to fetch, in groups of three to seven participants. - Dog and handler trained to AAS. Dog personality and aggressiveness evaluation. | Usual care in their respective settings. | 12 weeks after the intervention ended |
| Olsen (2) 2016 (Norway) | RCT | - Nursing home inpatients - Age: ≥65 years old, I 82.9 (SD 6.7); C 84.1 (SD 8.5) - Dementia: MMSE score<25, CDRS 2 a 3 (I 92% C 92.4%) - Inclusion: - - Exclusion: fear of dogs or with a dog allergy. | I: 25, C: 26 | - 30-min session twice weekly for 12 weeks. - Setting: nursing home residency. - Activities: Petting the dog, feeding the dog a treat and throwing a toy for the dog to fetch, in groups of three to six participants. - Dog and handler trained to AAS. Dog temperament tested. | Nursing Home care: diverse group activities such as reminiscence, music therapy, sensory garden, singing, exercise, cooking, and handicrafts. | 12 weeks after the intervention ended |
| Pope 2016 (USA) | Cross-over RCT | - Nursing home inpatients - Age: ≥65 years old, mean 79.8 (35 to 98 years old) - Dementia: Diagnosis of primary dementia, ranged from minor to severe. - Inclusion: having family agreement, verbal assent of the residents for each visit, and residents living at a health and rehabilitation center in a Southeastern state in the USA. - Exclusion: - | 44 in total | - 10 minutes visit, twice weekly for two weeks per intervention, with one break week. - Setting: nursing home residency. - Activities: interactions included touching, petting, brushing, holding, talking to and playing with the dogs, individually. - Dog and handler trained to AAS. Dog temperament was tested. | Human visit, interaction included general conversation and, reading from and looking at  pictures in a newspaper. | Immediately after the intervention ended |
| Bono 2015 (Italy) | RCT | - Outpatients - Age: ≥65 years old, I 82.1 (SD 6.2); C 78.3 (SD 10.3) - Dementia: MMSE scores 16 to 24, mean 17. 26 (SD 3.7). - Inclusion: If they had not been pet-owners or in contact with dogs during their life, if no general illness was evident or, in case of comorbidity, the disease was controlled by a therapy taken at home. - Exclusion: Use of concomitant specific drug therapies or alternative medicine interventions such as music therapy, shiatsu massage, etc. | I: 16, C: 16 | - One-hour sessions, twice weekly for 8 months. - Setting: a day-care center - Activities: Cognitive stimulation (dog “introduction” to the patient, identification of the dog by illustrations, learning basic commands, etc.), Communication (learning the body language of the animal, stimulating relationship with other participants), Motor activity (strolling with dog; petting the dog; throwing a small ball; grooming the dog; stimulation of manual skills), and Wellbeing and entertainment. - Dog and handler trained to AAS. Dog health and temperament was tested. | Usual care in their respective houses. | Immediately after the intervention ended |
| Friedman 2015 (USA) | RCT | - Assisted living facilities inpatients - Age: ≥55 years old, I 79.59 (SD 9.74); C 82.11 (SD 8.36) - Dementia: MMSE scores 8 to 23, mean 14.30 (SD 4.5). - Inclusion: anticipated length of stay in the AL facility for 6 or more months, English speaking, and with either prior experience with or interest in interacting with a dog. - Exclusion: Known allergies to or fear of dogs, a physical illness like asthma that is exacerbated in the presence of a dog, or receiving hospice care | I: 22, C: 18 | - 60 to 90-minute session, twice weekly for 12 weeks. - Setting: assisted living facilities. - Activities: Daily living (feeding the dog, brushing the dog’s teeth, brushing the dog’s hair, and dressing the dog in a bandana), Range of motion (throwing a ball, grooming the dog), Small motor skills (adjusting a collar, hand feeding a treat, and petting the dog), Sequencing events (opening a container of treats and then giving the dog a treat), and Social skills (talking to the dog, talking about the dog to another person, giving the dog commands, and petting the dog). - Dogs with experience at visiting a nursing home. | Reminiscence: Attentional control activities. Conversation related to a random topic and/or thing that had been happening in the residence. | Immediately after the intervention ended |
| Travers 2013 (Australia) | RCT | - Nursing home inpatients - Age: ≥65 years old, I 84.9 (SD 6.1); C 85.1 (SD 6.6) - Mild to moderate dementia registered in the medical record. Mean MSE-3MS: I 58.1(SD 19.8) C 59.8 (SD 17.2). - Inclusion: - - Exclusion: Severe dementia | I: 27 C: 28 | - 40 to 50 minutes session, three days a week, for 11 weeks. - Setting: nursing home residency. - Activities: interaction with the dog through play, petting and/or feeding it, and concluded by reading a short story to the group. - Dog and handler trained to AAS. Dogs were fully vaccinated and in good physical health. | Human visit adopted the same format but instead of a dog used the human interaction, it consists of a discussion with an article (unusual insect in a bottle, plant cutting). | Immediately after the intervention ended |
| Gallardo-Schall 2014 (Spain) | QE | - Nursing home inpatients - Age: ≥65 years old, I 84.9 (SD 6.1); C 85.1 (SD 6.6) - mean age of 87.96 years old - Dementia diagnostic registered in the medical record. Mean MMSE: I 9.63 C 10.63. - Inclusion: present behavioral and psychological symptoms of dementia. - Exclusion: -. - Control group allocation: no information | I: 11, C: 12 | - 35 minutes session, twice weekly for 8 weeks. - Setting: nursing home residency. - Activities: cognitive stimulation sessions with a dog (Basic obedience, signs of calm, tell a story, etc.). - A dog trained to AAS. | Usual care at the nursing home | Immediately after the intervention ended |
| Majíc 2013 (Germany) | QE | - Nursing home inpatients - Age: ≥55 years old, I 81.3 (SD 10.2); C 82.1 (SD 8.7). - Dementia: MMSE <25 and fulfilled the criteria for dementia of the DSM-IV. Mean MMSE: I 6.37 (SD 5.41) C 7.63 (SD 5.94). - Inclusion: duration of cognitive impairment was <6 months; and clinically significant cognitive impairment. - Exclusion: Delirium or other relevant Axis I diagnosis such as schizophrenia or bipolar disorder, or terminal somatic illness as defined by clinical examination and history taking. - Control group allocation: for convenience from the same nursing home. | I: 27, C: 27 | - 45 minutes sessions, weekly for 10 weeks. - Setting: nursing home residency. - Activities: Verbal interaction (patient speaking to the dog), -Physical interaction (stroking/petting the dog, Active interaction (throwing balls and retrieving them), and spontaneous interaction (the last 15 minutes). - Dogs trained to AAS. Periodically evaluated by a veterinarian. | Usual care at the nursing home (ongoing pharmacologic and nonpharmacologic treatment, including ergotherapy, massage, and physiotherapy) | Immediately after the intervention ended |
| Mossello 2011 (Italy) | QE | - Outpatients - Age: ≥65 years old, mean 79 years (SD 6). - Diagnosis of AD according to NINCDS-ADRDA criteria. Mean MMSE 3.3 (SD 5.5). - Inclusion: Attended the day-care center at least three times per week - Exclusion: - - Control group allocation: no information | 10 in total | - 100 minutes sessions, three times a week for two weeks. - Setting: a day-care center - Activities: an established sequence of actions with the dog, including talking, stroking, playing, feeding, brushing and taking for a short walk. - Dogs trained to AAS. Periodically evaluated by a veterinarian. | Same as intervention, but with plush-dog. | Immediately after the intervention ended |
| Kanamori 2001 (Japan) | QE | - Outpatients - Age: ≥65 years old, mean age 79.43 years old (SD 6.1) - Dementia: diagnosed by the DSM-IV criteria. Mean MMSE: I 11.4 (SD 9.9) C 10.2 (SD 7.0). - Inclusion: Those who gave consent - Exclusion: those who reported repulsion and fear towards animals - Control group allocation: for convenience from the same day-care center. | I: 7, C: 20 | - Bi-weekly sessions for 3 weeks. - Setting: a day-care center - Activities: daily activities to patient-dog interaction (no detailed). - Dogs trained to AAS. Periodically evaluated by a veterinarian. | Usual care at the day-care center. | 12 weeks after the intervention ended |

*** (I: intervention, C: control)**

**CDRS:** Clinical dementia rating scale; **MSE-3MS:** Modified Mini-Mental State Examination; **AD:** Alzheimer disease **NINCDS-ADRDA:** National Institute of Neurological and Communicative Disorders and Stroke and the Alzheimer's Disease and Related Disorders Association.
